# Uptake and metabolism of the antidepressants sertraline, clomipramine, and trazodone in a garden cress (*Lepidium sativum*) model

**DOI:** 10.1002/elps.201700482

**Published:** 2018-03-08

**Authors:** Bernd Reichl, Markus Himmelsbach, Lisa Emhofer, Christian W. Klampfl, Wolfgang Buchberger

**Affiliations:** ^1^ Institute of Analytical Chemistry Johannes Kepler University Linz Austria

**Keywords:** Environmental analysis, High performance liquid chromatography‐mass spectrometry, Plants, Xenobiotics

## Abstract

Environmental contamination with pharmaceuticals has received growing attention in recent years. Several studies describe the presence of traces of drugs in water bodies and soils and their impacts on nontarget organisms including plants. Due to these facts investigations of the uptake and metabolism of pharmaceuticals in organisms is an emerging research area. The present study demonstrates the analysis of three selected antidepressants (sertraline, clomipramine, and trazodone) as well as metabolites and transformation products in a cress model (*Lepidium sativum*). Cress was treated with tap water containing 10 mg/L of the parent drugs. Employing an analytical approach based on high performance liquid chromatography coupled with quadrupole time of flight or Orbitrap mass spectrometry in MS and MS² modes, in total 14 substances were identified in the cress extracts. All three parent drugs were taken up by the cress and translocated from the roots to the leaves in specific patterns. In addition to this, eleven metabolite species were identified. They were generated by hydroxylation, demethylation, conjugation with amino acids, or combinations of these mechanisms. Finally, the inclusion of control cultures in the experimental setup allowed for a differentiation of “true” metabolites generated by the cress and transformation products generated by plant‐independent mechanisms.

**Abbreviations**
ACN: acetonitrile
CLP: clomipramine
HPLC: high performance liquid chromatography
QTOF: quadrupole time of flight
STR: sertraline
TZN: trazodone

## 1 Introduction

Environmental contamination with pharmaceuticals has received growing attention in recent years. The presence of drugs in water bodies and soils has been reported in numerous studies 1, 2, 3, 4, 5, 6, 7, 8, 9. The main sources for these contaminants are therapeutic drug use in humans, therapeutic and preventive drug use in animals, pharmaceuticals used as growth promoters in livestock, and improper waste management in hospitals and pharmaceutical companies 6, 10. In an organism, pharmaceuticals are usually metabolized. A specific amount of the parent drug gets excreted alongside with its metabolites by the organism, entering domestic waste water. Depending on their persistence, pharmaceuticals and their metabolites are then more or less efficiently degraded during waste water treatment. Hence, residues of the original substances as well as their metabolites can potentially pass over to the waste water treatment effluent or to the sewage sludge. Both effluent and sewage sludge are reintroduced into the environment. Applying sewage sludge on farmland is common practice worldwide due to its nutritional and soil conditioning properties 4, 11. The waste water treatment effluent instead is either directed to surface water or it is directly applied on farmland for irrigation purposes, which is specifically the case in arid and semi‐arid regions like Israel, Spain, or California 5.

Pharmaceuticals have shown various impacts on nontarget organisms including plants. Antibiotics, for example, are reported to affect germination, growth, and development of plants 1. These drugs are extensively used in the treatment and prevention of diseases of livestock worldwide. Whereas the European Union banned antibiotics to be adopted as feed additive to promote animal growth, this practice is still very common in other areas. Other examples are endocrine disrupting chemicals like 17α‐ethinyl estradiol, a synthetic, highly active estrogen and the active substance in birth control pills. They have shown effects in various aquatic species, such as sexual development disruption and consequently distorted sex ratios 12. Increasing efforts in transcriptomic and proteomic analysis have revealed that besides physiological and biochemical impacts, the presence of xenobiotic compounds like pharmaceuticals can also lead to changes in signaling pathways 13. Apart from the effects single pharmaceuticals are reported to have on organisms, there is great concern about potential unwanted synergistic effects 3.

In order to assess the uptake and metabolism of pharmaceuticals in plants, sophisticated analytical techniques are required. Analysis of pharmaceuticals and their metabolites in plant extracts is predominantly done employing high performance liquid chromatography (HPLC) coupled to mass spectrometry (MS). Whereas known targets such as the parent drugs are usually analyzed with highly sensitive triple quadrupole (QQQ) instruments, the discovery of unknown analytes such as metabolites or transformation products requires high resolution mass analyzers such as quadrupole time of flight (QTOF) MS or Orbitrap MS instruments 2, 14.

Antidepressants are a subset of pharmaceuticals that are frequently found in the environment and show increasing numbers of prescription in recent years 6 (see also http://stats.oecd.org/index.aspx?DataSetCode=HEALTH_STAT#). A number of studies have been published describing the analysis of antidepressants in the aqueous environment 15, 16, 17, 18, 19. However, uptake and metabolization of antidepressants in plants is yet an understudied area.

**Figure 1:**
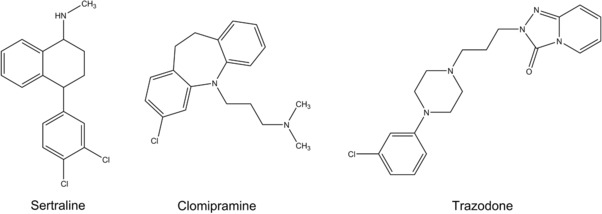
Structures of the three investigated antidepressants: sertraline, clomipramine, and trazodone.

In the present study, three widely used antidepressants – sertraline (STR), clomipramine (CLP), and trazodone (TZN) – were investigated (for chemical structures see Fig. 1). STR belongs to the group of selective serotonin reuptake inhibitors (SSRIs), TZN to the class of serotonin antagonists and reuptake inhibitors (SARIs), and CLP belongs to the class of tricyclic antidepressants (TCAs). The experimental work was directed toward gaining valuable information on the uptake of these three antidepressants in the model plant garden cress. Additionally, a focus was set on the identification of new metabolites formed within the plant species.

## 2 Materials and methods

### 2.1 Reagents and materials

TZN hydrochloride and CLP hydrochloride were purchased from Sigma Aldrich (Steinheim, Germany). Stock solutions with a concentration of 1000 mg/L were prepared by dissolving the respective amounts in 50% acetonitrile (ACN). STR (STR 50 mg, 1A Pharma) was obtained in the form of a pharmaceutical preparation. Tablets were homogenized with mortar and pestle and appropriate amounts were dissolved in 50% ACN to prepare 1000 mg/L stock solutions.

ACN (HPLC grade) and hydrochloric acid were purchased from Fisher Chemicals (Vienna, Austria), methanol (HPLC grade) and ethanol from VWR (Vienna, Austria). The mobile phase additives formic acid and ammonium formate were obtained from Sigma Aldrich. High purity water was prepared using a Milli‐Q water purification system (Millipore, Bedford, MA, USA).

Cress seeds (*Lepidium sativum*) were purchased from Austrosaat (Vienna, Austria). The seeds were irrigated with tap water from the university water system.

### 2.2 Plant cultivation and treatment

Garden cress was cultivated under hydroponic conditions. One and a half gram cress seeds were put into rectangular dishes (approximately 12 × 13 × 3.5 cm) upon a layer of paper towel which was humidified with 25 mL tap water. The dishes were then put into small greenhouses. After a germination period of 3 days treatment with the three selected antidepressants was started. The growing medium was obtained by diluting the antidepressant stock solutions in tap water, ending up at a final concentration of 10 mg/L of each antidepressant. Alongside with the treated cress cultures, a growing medium control sample (GM control) was generated by keeping the growing medium in a dish without garden cress from day 3 until 7 (day of harvest). This control was used to identify transformation products that were generated without any contribution of the plant's metabolism. In addition, a negative control was obtained by cultivating cress under similar conditions as the drug‐treated plants, but using pure tap water instead.

### 2.3 Preparation of plant extracts

The cress treatment was terminated on day 7 and plants including their roots were collected from the dishes. The upper parts of the plants were then separated from the roots allowing for an investigation of differences in the distribution of parent substances and metabolites within the plant. One gram of each separated plant and root material was then transferred into 15 mL polypropylene centrifuge tubes and washed three times by adding purified water, shaking the tubes, and discarding the water. Afterward 2 mL of ACN was added and the samples were thoroughly homogenized using an Ultra‐Turrax (Type TP18/10, Janke & Kunkel IKA‐Labortechnik, Staufen, Germany). After 15 min of incubation at ambient temperature the suspension was vortexed again and incubated for another 15 min, followed by a centrifugation at 4000 g for 3 min at ambient temperature. The GM control samples were directly transferred into HPLC vials. All samples were kept frozen at approximately −20°C until analysis.

### 2.4 HPLC‐MS and HPLC‐MS² instrumentation and data analysis

Analysis of the plant extracts was performed using reversed phase (RP) HPLC hyphenated with high resolution mass spectrometry. A modular Agilent 1100 HPLC system (with QTOF 6510 mass spectrometer) and an Agilent 1260 HPLC system (with Orbitrap XL mass spectrometer) consisting of a degasser, a quaternary pump, an autosampler, a column oven, and a diode‐array UV detector were used (Agilent Technologies, Waldbronn, Germany). Analytes were separated on a Kinetex C18 column (50 × 3 mm, particle size 2.6 μm, Phenomenex, Aschaffenburg, Germany) using a water/ACN gradient. The analytical column was protected by a C18 security guard column (4 × 3 mm, Phenomenex). Starting conditions were set to 5% solvent A (ACN), 75% solvent B (water), 10% solvent C (1% formic acid in ACN), and 10% solvent D (50 mM ammonium formate in water). Solvents C and D were kept constant at 10% each throughout the whole method. Solvent A was increased linearly to 30% within the first 10 min, then further increased to 80% within 1 min and kept constant for 7 min. Re‐equilibration to the starting conditions was achieved over a period of 5 min, resulting in a total run time of 23 min. The flow rate was set to 0.6 mL/min, the column was thermostated at 30°C, and an injection volume of 10 μL was used. Detection was carried out using either an Agilent 6510 QTOF (Agilent Technologies) or a Thermo Orbitrap XL (Thermo, Waltham, MA, USA) mass spectrometer, both equipped with an electrospray ionization (ESI) source and run in the positive ion mode. The QTOF was operated in full scan mode with a drying gas flow rate of 8 L/min, a drying gas temperature of 350°C, a nebulizer pressure of 45 psi, a capillary voltage of 3750 V, and a fragmentor voltage of 125 V. The Orbitrap was run with a sheath gas flow rate of 45 arbitrary units (au), an auxiliary gas flow rate of 15 au, a spray voltage of 3500 V, a capillary temperature of 350°C, a capillary voltage of 25 V, and a tube lens setting of 100 V. Mass resolution was set to 30 000. In MS^2^ analysis the Orbitrap was run in product ion scan mode, using collision induced dissociation to achieve fragmentation. Limits of detection (LOD) of both analytical methods were in the low microgram per liter range for the parent drugs with the Orbitrap analyzer showing slightly better LODs. The data were analyzed using Masshunter Workstation (Agilent) and XCalibur software (Thermo).

## 3 Results and discussion

In this study the uptake and metabolism of the antidepressants STR, CLP, and TZN in garden cress were investigated. Garden cress was chosen as model plant because of the possibility for hydroponic cultivation, eliminating the presence of potentially interfering compounds in soil. Short cycle times for growth, treatment, harvest, extraction, and analysis (usually 7 days) are a further advantage of this model plant. In order to identify potential metabolites, the cress was treated with a concentration of 10 mg/L antidepressants in tap water. Despite such concentrations being very unlikely to occur in the natural environment, this concentration range has been regularly used in similar studies 20, 21, 22, 23, 24, 25, 26, 27, 28.

### 3.1 Distribution of parent drugs

The first goal in this study was to investigate the uptake and distribution of the parent drugs in the model plant. The experimental setup comprised the cultivation and treatment of cress in three replicates. Extracts of the separated plant parts as well as the growing medium (GM) control sample were analyzed by HPLC‐MS (QTOF) in full scan mode. The three parent drugs showed signals of their protonated molecular ion [M + H]^+^ and were identified based on their exact masses and by comparing retention times with standard substances. Quantification was done using external calibration. In addition to the protonated precursor, STR showed a fragment ion with a mass to charge ratio (*m*/*z*) of 275.0, which is described as typical in‐source fragment that evolves after a neutral loss of CH_3_NH_2_ 29, 30. Quantitative results show that the three parent substances STR, CLP, and TZN are taken up and translocated from the roots to the upper part of the plants in substance‐specific patterns (Table 1). These observations comply with the fact that organic substances are taken up by plants mainly via plant transpiration, which is a passive uptake process of water by roots and its translocation to leaves 31, 32. More polar compounds like TZN are therefore more likely to be translocated to the leaves compared to apolar compounds. Relative standard deviations in this experimental setup (*n* = 3) were between 14 (TZN in leaves) and 27% (CLP in root). Despite these variances being acceptable for a biological model, the employment of replicate cultures should always be considered.

**Table 1:** Comparison of average concentrations (in μg/g plant material) of the three parent drugs in treated root and leaf samples based on three cress culture replicates

	Roots			Leaves		
	(μg/g)	SD	RSD (%)	(μg/g)	SD	RSD (%)
Sertraline	4.29	0.98	23	0.52	0.14	26
Clomipramine	1.83	0.50	27	1.14	0.25	22
Trazodone	0.91	0.15	17	2.63	0.37	14

### 3.2 Screening for metabolites

The second goal in this study was the identification of metabolites in the treated cress samples. Since no standards for metabolites were available in this study allowing for a comparison of retention times, identification of metabolites was only possible based on their exact masses and fragmentation patterns. The first approach was to screen the acquired MS full scan data for metabolites reported in the literature studying metabolism of the parent drugs in human and animal models 33, 34, 35, 36, 37, 38, 39, 40. This approach was selected because plants used in biological wastewater treatment such as constructed wetlands were reported to transform organic compounds to similar metabolites like humans or animals do 2, 31, 41. These metabolites are, for example, methylated, demethylated, hydroxylated, conjugated, or other derivatives of the parent drugs. This literature research resulted in a list of 21 metabolite candidates (see Supporting Information Table 1). The analytical setup employed in this series of experiments comprised two high resolution MS instruments (QTOF in full scan mode and Orbitrap XL in full scan and MS² product ion scan mode). An overview of the detected parent drugs and related metabolites is given in Table 2. Due to the fact that the ion‐trap part of the Orbitrap allows ion accumulation, superior sensitivity was achieved with this instrument. This allowed the singular detection of three metabolites (Table 1) that were only present in minor concentrations. On the other side, sensitive detection with the Orbitrap XL required careful optimization of trap related parameters. For this reason, when a first screening for metabolites was performed, the QTOF instrument was preferred as it could be used with generic parameters and still provided sufficient sensitivity for the identification of the major metabolites.

**Table 2:** Parent drugs and their metabolites detected in plant root extract from cress treated with 10 mg/L of the antidepressants. MS data (except for^a)^) measured with QTOF MS, MS^2^ data measured with Orbitrap XL

Compound	Sum formula	RT	*m*/*z* (theor.)	*m*/*z* (meas.)	Mass error/ppm	Measured product ions^b)^	Normalized collision energies^b)^	Activation time/ms^b)^
STR	C_17_H_17_Cl_2_N	10.2	306.0811	306.0823	3.9	275.0	14	20
OH‐STR (isomer 1)	C_17_H_17_Cl_2_NO	6.7	322.0760	322.0762	0.6	273.0	13	30
OH‐STR (isomer 2)	C_17_H_17_Cl_2_NO	8.2	322.0760	322.0763	0.9	273.0	13	30
DM‐STR + Phe^a)^	C_25_H_24_Cl_2_N2O	13.1	439.1330	439.1332	0.5	275.0/165.1/91.1	20	30
DM‐STR + Tyr^a)^	C_25_H_24_Cl_2_N_2_O_2_	12.7	455.1290	455.1281	−2.0	275.0/181.1/107.1	20	30
OH‐DM‐STR + Phe^a)^	C_25_H_24_Cl_2_N_2_O_2_	12.5	455.1290	455.1281	−2.0	273.0/165.1	20	30
CLP	C_19_H_23_ClN_2_	10.7	315.1623	315.1638	4.8	270.1/242.1/86.1	19	50
DM‐CLP	C_18_H_21_ClN_2_	10.4	301.1466	301.1469	1.0	270.1/242.1/72.1	17	30
OH‐CLP (isomer 1)	C_19_H_23_ClN_2_O	6.8	331.1572	331.1584	3.6	313.2/86.1	14	30
OH‐CLP (isomer 2)	C_19_H_23_ClN_2_O	7.0	331.1572	331.1582	3.0	313.2/86.1	14	30
OH‐DM‐CLP	C_18_H_21_ClN2O	6.6	317.1415	317.1421	1.9	299.1/86.1	11	40
TZN	C_19_H_22_ClN_5_O	5.5	372.1586	372.1597	3.0	176.1/148.1	20	40
OH‐TZN	C_19_H_22_ClN_5_O_2_	6.3	388.1535	388.1540	1.3	176.1/148.1	20	30
mCPP	C10H_13_ClN_2_	3.0	197.0840	197.0843	1.5	154.0	26	30

Only observed with Orbitrap XL.

Measured with Orbitrap XL.

Among the STR metabolites, hydroxylated STR (OH‐STR) was found in both root and leaf extracts at a retention time (RT) of 6.7 min. The compound shows a *m*/*z* ratio of 322.0762, the typical dichloro isotopic pattern (9:6:1) and upon product ion scan in the Orbitrap a fragment with *m*/*z* 273.0. This observation agrees with a previously described fragmentation pattern found when STR was added to different soils 30. The generation of this fragment was explained by the neutral loss of CH_3_NH_2_ and H_2_O, and the existence of an additional double bond in the nonaromatic ring. This OH‐STR derivative was not only present in plant extracts, but also in traces in the GM control sample, which was the growing medium only, kept in a culture dish from day 3 until 7 without garden cress. This control sample was used to identify transformation products that were generated without any contribution of the plant´s metabolism. Therefore, the presence of OH‐STR in the GM control suggests that there is also a plant‐independent transformation mechanism. In order to assess the contribution of both mechanisms, namely plant's metabolism and plant‐independent transformation reactions, the ratios of the metabolite's integrated peak areas in the root extract and the GM control sample were calculated. A high ratio would indicate a metabolite generation primarily in the plant, whereas a low ratio would rather suggest a transformation outside of the plant and subsequent uptake into the plant tissue. In the case of OH‐STR this ratio is 51. Such a high ratio could either mean that the compound is already generated in the growing medium and then taken up by the cress highly efficiently, or, more likely, that it is generated both by plant‐independent transformation as well as metabolism in the cress. A second compound with *m*/*z* 322.0763 and a dichloro isotopic pattern was found at RT 8.2 min. This derivative is regarded to be a structural isomer, having the OH group attached at a different position. It is only present in the roots and in very low abundances in the leaves, but it was not detected in the GM control sample at all, indicating that this isomer is a true STR metabolite.

In the case of the second selected antidepressant CLP, two isomers with *m*/*z* 331.1584 and 331.1582 were found (RTs 6.8 and 7.0 min, respectively). These compounds were allocated to hydroxylated CLP (OH‐CLP) based on their exact mass and a characteristic fragment with *m*/*z* 86.1 which they have in common with the parent CLP molecule. This fragment is reported to be generated when the bond between the central nitrogen atom and the C_5_H_12_N chain breaks, leaving the charge on the chain 42. An additional fragment with *m*/*z* 313.2 was observed in both isomers, reflecting a water loss of the metabolite. An 8‐OH‐CLP was reported before 38, but the fact that our results show two compounds with the same *m*/*z* values and fragmentation patterns suggest the existence of a further isomer, in which the hydroxyl group is located at another position. Focusing on the question if these hydroxylated derivatives were generated by the cress or by other mechanisms, it must be noted that both isomers were present in very high abundances in the root and also in leaves extracts, but only in traces in the GM control. The integrated peak areas in root normalized to the GM control (378 and 491, respectively) suggest that both OH‐CLP isomers are true metabolites and only a very small contribution can be attributed to a plant‐independent transformation mechanism already in the GM. In addition to the OH‐CLP derivatives two further metabolites were found, namely demethyl‐CLP (DM‐CLP, *m*/*z* 301.1469, RT 10.4 min) and hydroxy‐demethyl‐CLP (OH‐DM‐CLP, *m*/*z* 317.1421, RT 6.6 min). In product ion scan using Orbitrap DM‐CLP shows fragments with *m*/*z* 270.1 and 242.1 (similar to the parent drug) as well as a fragment with *m*/*z* 72.1. OH‐DM‐CLP shows a fragment with *m*/*z* 299.1, reflecting a water loss, as well as a fragment with *m*/*z* 86.1, which it has in common with the parent drug. DM‐CLP is present in root and leaves, but also in the GM control, showing a ratio between root and control of 1.84. Compared with the OH‐CLP isomers described in this section, an interpretation if the compound is a true metabolite or not is more difficult. Since the ratio between root and negative control lies in between the previously described ones, it is suggested that DM‐CLP is both a transformation product as well as a metabolite generated by the cress. OH‐DM‐CLP instead was present in cress extracts only, but not in the GM control, indicating that this compound is a true metabolite.

**Figure 2:**
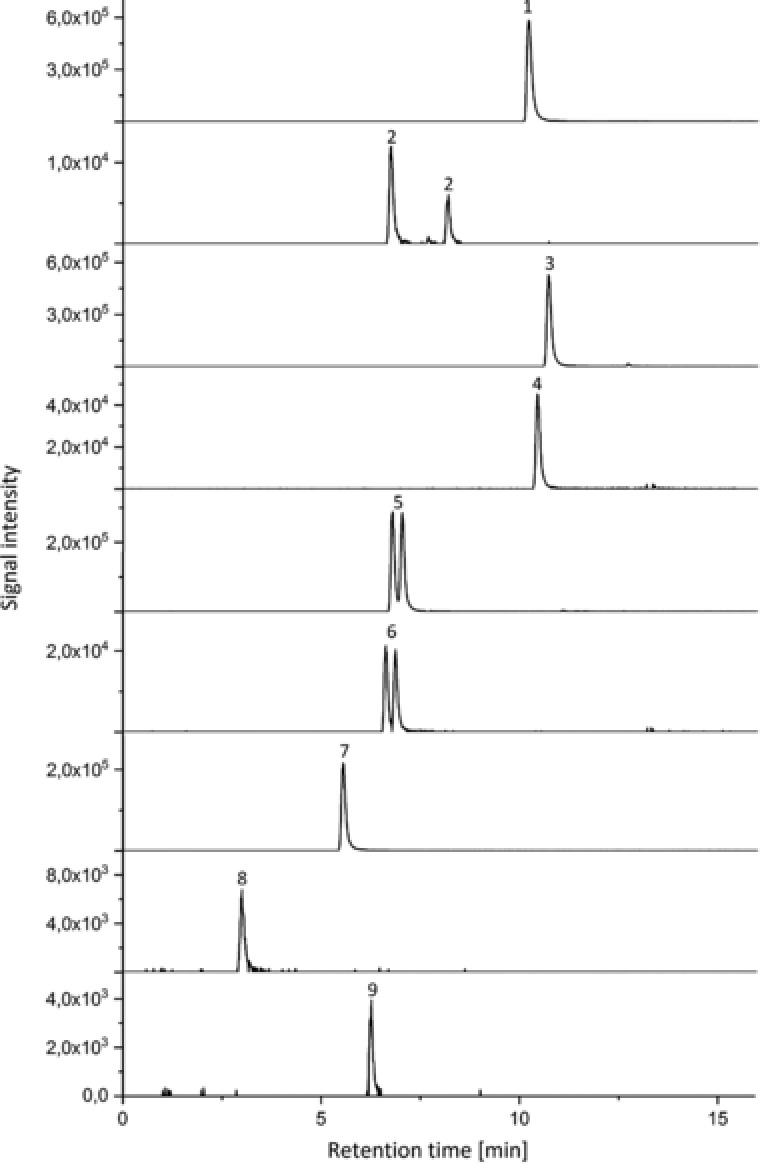
Extracted ion chromatograms (QTOF) obtained for a root extract from cress treated with 10 mg/L of the antidepressants. Peaks: 1: STR; 2: OH‐STR (two isomers); 3: CLP; 4: DM‐CLP; 5: OH‐CLP (two isomers); 6: OH‐DM‐CLP (two isomers); 7: TZN; 8: mCPP; 9: OH‐TZN. For conditions please see Section 2.

With regards to TZN, its pharmaceutically active metabolite meta‐chlorophenylpiperazine (mCPP, *m*/*z* 197.0843, RT 3.0 min) was detected in root and leaf samples, but also in traces in the control. With a ratio between root and negative control of 25 it is suggested to be both metabolite and transformation product. Product ion scan using Orbitrap resulted in a fragment with *m*/*z* of 154.0. In addition to mCPP, a hydroxylated derivative of TZN (OH‐TZN, *m*/*z* 388.1540, RT 6.3 min) was detected in the root extracts, showing the same fragments as the parent TZN molecule (*m*/*z* 176.1 and 148.1). Figure 2 shows extracted ion chromatograms (HPLC‐QTOF) obtained for a plant root extract from cress treated with antidepressants at a concentration of 10 mg/L each. As can be seen from this figure the three parent drugs could be detected together with six proposed metabolites (several of them showing structural isomers).

Whereas parent drugs and their metabolites were present in plant samples and the GM control, the negative control (cress cultivated in tap water) was negative for all investigated compounds.

### 3.3 Untargeted screening

**Figure 3:**
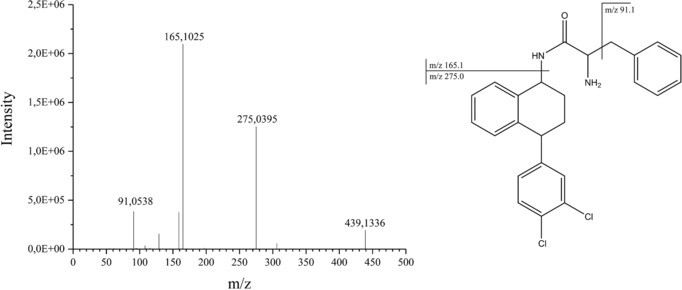
MS^2^ spectrum of DM‐STR conjugated with phenylalanine obtained with Orbitrap MS in product ion scan mode using a precursor isolation width of 1 u.

**Figure 4:**
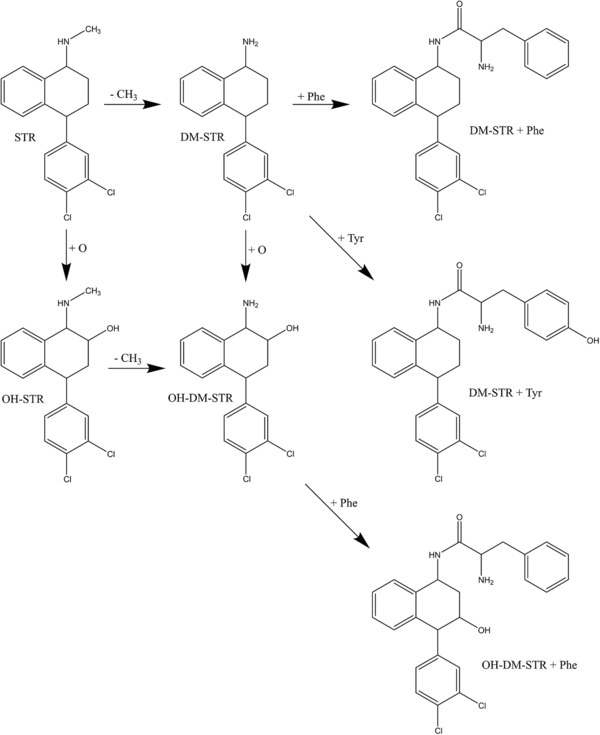
Proposed structural formulas (actual position of substitution not known exactly) and proposed scheme of the formation of STR metabolites.

**Figure 5:**
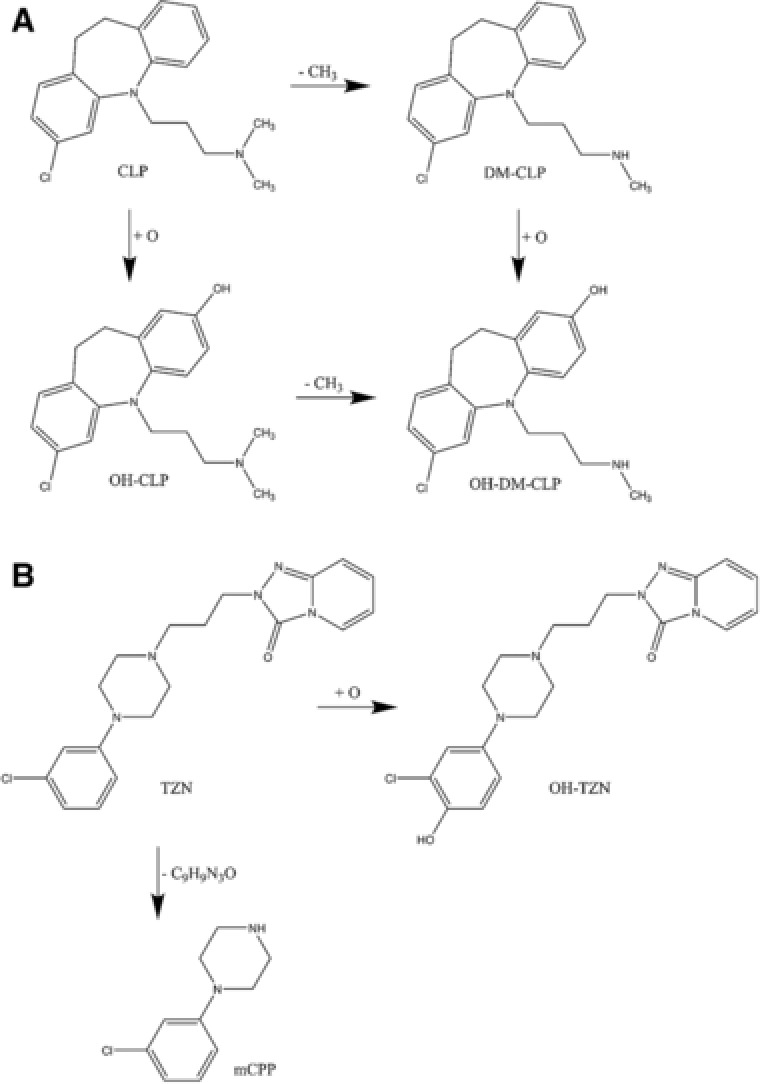
Proposed structural formulas (actual position of substitution not known exactly) and proposed scheme of the formation of CLP (A) and TZN (B) metabolites.

In addition to the approach described in section 3.2, the data were screened for additional analytes comprising the typical chlorine isotopic patterns (3:1 for compounds with one chlorine atom or 9:6:1 for two chlorine atoms). The identification of the unknown compounds was based on their exact masses and fragmentation patterns. In this approach, the Orbitrap mass spectrometer was used exclusively due to its better sensitivity compared to QTOF, after ion source parameters like capillary temperature, capillary voltage, or tube lens were optimized. First, a compound with *m*/*z* of 439.1332, RT 13.1 min and showing a dichloro isotopic pattern of 9:6:1 is suggested to represent demethyl‐STR conjugated with phenylalanine (DM‐STR‐Phe). The compound shows a fragment with *m*/*z* of 275.0 which it has in common with the parent STR molecule, as well as a fragment with *m*/*z* of 165.1, corresponding to the conjugated phenylalanine (see also Fig. 3 for the MS^2^ spectrum of DM‐STR‐Phe). In addition, two further STR‐derived metabolites (both with *m*/*z* 455.1281 and retention times of 12.5 and 12.7 min, respectively) are suggested to represent demethyl‐STR conjugated with tyrosine (DM‐STR‐Tyr) and hydroxy‐demethyl‐STR conjugated with phenylalanine (OH‐DM‐STR‐Phe). The first one (DM‐STR‐Tyr) shows the typical STR fragment with *m*/*z* of 275.0 as well as a fragment with *m*/*z* of 181.1, corresponding to the conjugated tyrosine rest. The latter one (OH‐DM‐STR‐Phe) shows the same fragment as OH‐STR (*m*/*z* = 273.0) as well as the fragment corresponding to the phenylalanine rest (*m*/*z* = 165.1) which was also observed in DM‐STR‐Phe as described in this section. With these findings STR shows the highest number of metabolites among the investigated antidepressants. In two figures proposed metabolization schemes of STR (Fig. 4), CLP (Fig. 5A) and TZN (Fig. 5B) leading to the observed metabolites are presented.

## 4 Concluding remarks

The present work describes the screening for metabolites in garden cress samples treated with three antidepressants employing high resolution mass spectrometers. Results show that the parent drugs are taken up by the model plant and translocated in substance‐specific patterns. A total of 11 metabolites were detected, 8 of them being reported before in human or animal models. Additionally, three conjugates with amino acids were detected. These findings provide valuable information on ways of drug metabolization in plants and build the basis for further investigations, for example, the development of an MRM method employing highly sensitive QQQ instruments.

*The authors have declared no conflict of interest*.

## Supporting information

Supporting information
