# Supplementary material for: Uptake and metabolism of the antidepressants sertraline, clomipramine, and trazodone in a garden cress (Lepidium sativum) model
Source: Electrophoresis. 2018 Mar 8;39(9-10):1301–8. doi: 10.1002/elps.201700482 (PMC6099436; doi:10.1002/elps.201700482)
Supplement: Supplementary file 1 — Supporting information [file ELPS-39-1301-s001.docx]

Table S1

| Parent drug/Metabolite | Sum Formula | M [+H] | Reference |
| --- | --- | --- | --- |
| Sertraline | C_17_H_17_NCl_2_ | 306.0811 |  |
| N-Demethylsertraline | C_16_H_15_NCl_2_ | 292.0654 | 34, 35 |
| 4-(3,4-Dichlorophenyl)-1-tetralone (= Sertraline-ketone) | C_16_H_12_OCl_2_ | 291.0338 | 35 |
| Sertraline-N-carbamoyl-glucuronide | C_24_H_25_NO_8_Cl_2_ | 526.1030 | 34, 35 |
| 4-(3,4-Dichlorophenyl)-2-hydroxy-1-tetralone | C_16_H_12_O_2_Cl_2_ | 307.0287 | 34 |
| N-Hydroxysertraline | C_17_H_17_Cl_2_NO | 322.0760 | 34 |
| Clomipramine | C_19_H_23_ClN_2_ | 315.1623 |  |
| N-Demethylclomipramine | C_18_H_21_ClN_2_ | 301.1466 | 37, 38 |
| N-Didemethylclomipramine | C_17_H_19_ClN_2_ | 287.1310 | 38 |
| 8- or 2-Hydroxyclomipramine | C_19_H_23_ClN_2_O | 331.1572 | 37, 38 |
| 8-Hydroxydemethylclomipramine | C_18_H_21_ClN_2_O | 317.1415 | 34, 37, 38 |
| 8-Hydroxyclomipramine-glucuronide | C_25_H_31_ClN_2_O_8_ | 523.1842 | 37, 38 |
| 8-Hydroxy-N-demethylclomipramine-glucuronide | C_24_H_29_ClN_2_O_8_ | 509.1685 | 34, 37, 38 |
| Clomipramine-N-oxide | C_19_H_23_ClN_2_O | 331.1572 | 34 |
| 8-Hydroxydidemethylclomipramine | C_17_H_19_ClN_2_O | 303.1259 | 34 |
| Trazodone | C_19_H_22_ClN_5_O | 372.1586 |  |
| meta-Chlorphenylpiperazine | C_10_H_13_ClN_2_ | 197.0840 | 33, 34, 36 |
| 4-Hydroxytrazodone | C_19_H_22_ClN_5_O_2_ | 388.1535 | 33, 34, 40 |
| Trazodone-N-oxide | C_19_H_22_ClN_5_O_2_ | 388.1535 | 39 |
| Trazodonedihydrodiol | C_19_H_26_ClN_5_O_3_ | 408.1797 | 34 |
| 4-Hydroxytrazodone-β-D-glucuronide | C_25_H_30_ClN_5_O_8_ | 564.1856 | 34 |
| Trazodonedihydrodiol-N-oxide | C_19_H_26_ClN_5_O_4_ | 424.1746 | 34 |
| Triazolopropionic acid | C_9_H_9_N_3_O_3_ | 208.0717 | 36 |
| 4-Hydroxy-meta-chlorophenylpiperazine | C_10_H_13_ClN_2_O | 213.0789 | 34 |
